# Supplementary material for: A Likelihood Approach for Real-Time Calibration of Stochastic Compartmental Epidemic Models
Source: PLoS Comput Biol. 2017 Jan 17;13(1):e1005257. doi: 10.1371/journal.pcbi.1005257 (PMC5240920; doi:10.1371/journal.pcbi.1005257)
Supplement: S1 File — (TAR.GZ) [file pcbi.1005257.s014.tar.gz › HSPH_Online-SI-Revision/Additions/PF09/n100-extreme_noisy/n100-extreme_noisy_PF_table-8.pdf]

1	R0	{2.76052, 16.3913}	{1.8755, 19.2326}	{{1.72642, 18.9189}, {2.08506, 20.}}	{32.06, 17.9828}	inf	695.	1143.39	{938.498, 1385.17}	64.5162	0.21081	newly	289.367	489.94	{383., 614.}	69.3143	0.221514	Reff	2.73137	1.84584	{1.70942, 2.05227}	32.4208	ar	92 090.	79 604.3	{74 728., 85 336.}	13.5582
2	R0	{2.18202, 15.1734}	{1.85414, 19.3287}	{{1.64839, 16.1985}, {2.00279, 20.}}	{15.0265, 28.4251}	inf	2011.	1696.8	{1387.55, 2351.05}	20.5947	0.10063	newly	1160.	710.45	{565., 935.}	38.7543	0.219949	Reff	2.10076	1.79452	{1.59965, 1.88538}	14.5775	ar	84 264.	79 204.8	{70 941., 83 810.}	6.34801
3	R0	{2.57891, 12.5971}	{1.8838, 18.1172}	{{1.62575, 14.8796}, {2.13953, 20.}}	{27.4146, 44.21}	inf	340.	473.434	{263.573, 605.709}	46.8958	0.159072	newly	303.	221.63	{114., 313.}	32.9604	0.189584	Reff	2.5648	1.86356	{1.61853, 2.12236}	27.7454	ar	90 302.	77 645.5	{68 648., 85 933.}	14.374
4	R0	{2.5856, 9.28508}	{2.11311, 15.5691}	{{1.39677, 8.23171}, {3., 20.}}	{24.0928, 72.2771}	inf	8.	225.499	{84.8109, 380.552}	2718.73	1.36521	newly	20.	158.68	{43., 373.}	693.5	0.753015	Reff	2.58508	2.08767	{1.39064, 2.9596}	24.5407	ar	90 312.	77 453.5	{52 612., 95 125.}	16.4006
5	R0	{2.4406, 12.1341}	{1.89046, 17.6388}	{{1.54495, 13.0792}, {2.30125, 20.}}	{24.1195, 47.128}	inf	184.	364.924	{204.704, 644.958}	98.9088	0.267716	newly	170.	178.99	{81., 301.}	36.1941	0.154251	Reff	2.43279	1.86448	{1.49434, 2.28381}	24.8271	ar	88 597.	75 858.5	{62 427., 88 173.}	15.3186
6	R0	{2.72155, 15.4094}	{1.84245, 18.9649}	{{1.62668, 15.5563}, {2.10261, 20.}}	{32.3015, 24.9262}	inf	411.	527.398	{330.241, 796.873}	37.1917	0.128393	newly	302.	234.43	{126., 390.}	35.1026	0.194268	Reff	2.7028	1.82179	{1.61874, 2.06448}	32.5963	ar	91 682.	77 198.1	{69 606., 85 121.}	15.7979
7	R0	{2.63781, 14.3935}	{1.79529, 19.9376}	{{1.73846, 20.}, {1.8446, 20.}}	{31.9403, 38.5183}	inf	448.	669.967	{561.288, 795.085}	49.5463	0.170369	newly	307.	269.55	{215., 334.}	16.2378	0.0785641	Reff	2.61787	1.78318	{1.72749, 1.83211}	31.8842	ar	91 192.	77 453.2	{75 411., 79 628.}	15.0658
8	R0	{2.82391, 16.104}	{1.85129, 18.8185}	{{1.58151, 15.5454}, {2.1182, 20.}}	{34.4424, 19.3449}	inf	560.	578.511	{367.361, 806.9}	26.0106	0.112644	newly	385.	255.07	{145., 360.}	35.0675	0.209085	Reff	2.79954	1.81878	{1.57272, 2.04875}	35.0328	ar	92 664.	77 120.7	{66 238., 85 584.}	16.7739
9	R0	{2.80645, 14.6457}	{1.79415, 19.5238}	{{1.63808, 18.41}, {2.02544, 20.}}	{36.0706, 33.5543}	inf	226.	465.238	{322.937, 689.46}	105.858	0.297503	newly	148.	194.79	{128., 304.}	37.4257	0.123161	Reff	2.79573	1.78002	{1.62878, 1.98154}	36.3309	ar	92 567.	75 821.6	{69 601., 82 867.}	18.09
10	R0	{1.86213, 5.4271}	{1.99953, 16.1223}	{{1.35208, 10.0662}, {2.75914, 20.}}	{25.0804, 197.07}	inf	32.	242.409	{82.7709, 389.226}	657.529	0.810911	newly	74.	137.23	{44., 222.}	101.446	0.288418	Reff	1.8607	1.97495	{1.31731, 2.74696}	24.6843	ar	75 362.	73 607.8	{45 451., 93 292.}	19.7002
11	R0	{2.27349, 14.6338}	{1.95038, 18.7122}	{{1.59611, 14.5445}, {2.19619, 20.}}	{14.2124, 28.8473}	inf	1278.	1336.93	{888.377, 1764.88}	23.4698	0.100546	newly	843.	591.46	{395., 743.}	29.9786	0.166889	Reff	2.22036	1.89597	{1.57739, 2.13056}	14.6206	ar	85 805.	81 595.5	{68 875., 88 763.}	7.18342
12	R0	{2.77261, 14.1894}	{1.79825, 19.9819}	{{1.73574, 20.}, {1.84854, 20.}}	{35.1423, 40.8225}	inf	206.	548.856	{457.949, 626.481}	166.435	0.421435	newly	178.	219.51	{173., 257.}	24.5225	0.0913613	Reff	2.76385	1.79226	{1.73178, 1.8419}	35.1535	ar	92 508.	77 591.4	{75 113., 79 798.}	16.1247
13	R0	{2.65402, 19.3935}	{1.82506, 19.9731}	{{1.71642, 20.}, {1.89427, 20.}}	{31.2342, 2.99245}	inf	1354.	1036.33	{869.098, 1257.35}	23.7057	0.121597	newly	658.	415.06	{342., 515.}	36.921	0.20555	Reff	2.59651	1.80062	{1.69038, 1.86596}	30.6524	ar	91 184.	78 766.	{74 016., 81 569.}	13.6187
14	R0	{1.75297, 4.45354}	{1.83361, 17.805}	{{1.46147, 12.8049}, {2.58594, 20.}}	{15.4304, 299.795}	inf	18.	324.739	{168.997, 515.778}	1704.11	1.21666	newly	29.	157.44	{63., 261.}	442.897	0.676227	Reff	1.75187	1.80957	{1.422, 2.57269}	15.1041	ar	71 263.	73 103.8	{57 262., 91 838.}	13.24
15	R0	{1.99928, 7.1501}	{1.89669, 17.7237}	{{1.49906, 14.0821}, {2.60658, 20.}}	{16.1781, 147.88}	inf	97.	355.997	{194.242, 593.845}	267.008	0.522253	newly	168.	173.29	{77., 270.}	36.2202	0.157925	Reff	1.99548	1.87574	{1.493, 2.58242}	16.5308	ar	79 602.	75 010.1	{60 188., 92 664.}	12.1015
16	R0	{2.67398, 10.3134}	{1.89328, 18.2832}	{{1.54609, 14.0213}, {2.56291, 20.}}	{29.4606, 77.7392}	inf	44.	328.769	{168.821, 554.687}	647.202	0.834357	newly	54.	154.13	{65., 274.}	185.648	0.404773	Reff	2.67219	1.86552	{1.50442, 2.54744}	30.4117	ar	91 289.	75 835.8	{63 269., 91 939.}	17.3722
17	R0	{2.44634, 16.3226}	{1.81788, 19.8643}	{{1.72, 19.98}, {1.87683, 20.}}	{25.6896, 22.1767}	inf	1288.	1006.98	{835.383, 1175.83}	21.8182	0.110275	newly	777.	409.91	{335., 483.}	47.2445	0.282161	Reff	2.39443	1.79391	{1.69659, 1.85002}	25.0799	ar	88 710.	78 449.5	{74 823., 81 120.}	11.5663
18	R0	{2.02168, 10.1676}	{1.80352, 19.8062}	{{1.74557, 20.}, {1.8836, 20.}}	{10.791, 94.7975}	inf	354.	682.333	{541.208, 823.806}	92.7495	0.279866	newly	351.	279.42	{213., 345.}	22.433	0.114776	Reff	2.00733	1.79102	{1.73592, 1.86898}	10.7757	ar	80 299.	77 689.4	{75 773., 80 845.}	3.56113
19	R0	{2.89969, 15.009}	{1.80197, 19.5616}	{{1.65396, 19.0804}, {2.05664, 20.}}	{37.8564, 30.7096}	inf	224.	442.14	{303.262, 689.291}	97.3841	0.276834	newly	179.	184.72	{122., 311.}	27.9106	0.112481	Reff	2.88972	1.78927	{1.64727, 1.98475}	38.0813	ar	93 349.	76 125.9	{70 824., 83 351.}	18.4502
20	R0	{2.68933, 7.34928}	{2.00965, 15.4565}	{{1.36435, 9.87798}, {2.95315, 20.}}	{28.2122, 110.313}	inf	3.	216.245	{64.7351, 406.923}	7108.18	1.76442	newly	7.	133.04	{34., 270.}	1800.57	1.16688	Reff	2.68909	1.98074	{1.36328, 2.92096}	28.6943	ar	91 574.	73 941.	{48 164., 94 816.}	20.3979
21	R0	{2.15352, 9.28074}	{1.8842, 17.3561}	{{1.45947, 12.9729}, {2.59908, 20.}}	{20.7745, 87.0631}	inf	131.	343.531	{206.335, 617.667}	162.237	0.385854	newly	151.	168.6	{78., 283.}	36.3576	0.147893	Reff	2.14827	1.86613	{1.44931, 2.57062}	21.102	ar	83 845.	74 188.9	{57 938., 92 271.}	15.711
22	R0	{2.21953, 9.04387}	{1.94801, 17.4492}	{{1.50024, 11.6213}, {2.59477, 20.}}	{19.6133, 93.2636}	inf	114.	278.491	{154.211, 439.296}	144.291	0.35472	newly	136.	143.84	{62., 244.}	39.7941	0.175685	Reff	2.21565	1.92534	{1.4892, 2.56718}	20.3283	ar	84 856.	76 645.3	{60 631., 92 905.}	13.4607
23	R0	{2.21987, 14.5347}	{1.8145, 19.9874}	{{1.7423, 20.}, {1.86736, 20.}}	{18.2612, 37.5154}	inf	1324.	1286.73	{1079.12, 1505.99}	11.3881	0.0503243	newly	822.	515.64	{411., 625.}	37.4526	0.208268	Reff	2.16759	1.77714	{1.71111, 1.82529}	18.0131	ar	85 067.	78 164.3	{75 448., 80 306.}	8.11443
24	R0	{2.84268, 17.9659}	{1.79344, 20.}	{{1.7594, 20.}, {1.82811, 20.}}	{36.9103, 11.322}	inf	799.	1031.56	{931.476, 1189.25}	29.1064	0.10918	newly	439.	411.99	{361., 483.}	10.4806	0.0477457	Reff	2.80769	1.76614	{1.73397, 1.80083}	37.0964	ar	92 836.	77 344.5	{76 041., 78 923.}	16.687
25	R0	{2.52706, 10.3679}	{1.80754, 19.9852}	{{1.73182, 20.}, {1.87562, 20.}}	{28.4726, 92.7601}	inf	97.	522.884	{412.204, 645.7}	439.056	0.725156	newly	97.	210.91	{163., 265.}	117.433	0.329397	Reff	2.52279	1.7999	{1.72784, 1.86558}	28.6543	ar	89 623.	77 717.5	{74 858., 80 350.}	13.284
26	R0	{2.62835, 19.1088}	{1.82281, 19.885}	{{1.70981, 19.9747}, {1.89335, 20.}}	{30.648, 4.90461}	inf	1699.	1085.07	{914.49, 1252.24}	36.7491	0.201212	newly	814.	438.08	{358., 518.}	46.5528	0.276252	Reff	2.55757	1.79554	{1.68639, 1.86533}	29.795	ar	90 908.	78 568.1	{74 419., 81 266.}	13.574
27	R0	{2.76831, 19.4155}	{2.00976, 18.4777}	{{1.66607, 15.3998}, {2.15812, 20.}}	{27.4012, 7.63287}	inf	2186.	1602.13	{1142.3, 1954.31}	26.7095	0.141557	newly	1084.	732.16	{565., 921.}	32.5221	0.178673	Reff	2.67358	1.92555	{1.63618, 2.07866}	27.9787	ar	92 175.	82 233.5	{71 077., 86 324.}	10.7854
28	R0	{2.96609, 16.8903}	{2.03072, 17.3827}	{{1.51557, 9.46836}, {2.65701, 20.}}	{31.6268, 18.9095}	inf	591.	445.135	{263.71, 723.217}	36.1255	0.20317	newly	383.	225.14	{115., 348.}	43.6031	0.283453	Reff	2.94132	2.00344	{1.50297, 2.64052}	31.9618	ar	94 024.	78 334.2	{61 242., 93 181.}	16.8604
29	R0	{2.17046, 14.39}	{1.81253, 19.9781}	{{1.72021, 20.}, {1.87581, 20.}}	{16.4912, 38.8333}	inf	1169.	1245.64	{1024.51, 1456.1}	12.129	0.0498661	newly	743.	501.19	{411., 586.}	32.5639	0.175134	Reff	2.12193	1.778	{1.68474, 1.83657}	16.2084	ar	84 001.	78 254.5	{74 706., 80 731.}	6.84104
30	R0	{2.88772, 12.4647}	{2.04299, 15.0343}	{{1.37437, 8.43154}, {2.98292, 20.}}	{30.2078, 33.484}	inf	43.	213.88	{69.3191, 393.029}	397.395	0.610444	newly	59.	151.93	{44., 314.}	167.373	0.327804	Reff	2.88584	2.01227	{1.36928, 2.95668}	31.0423	ar	93 273.	74 543.2	{48 766., 94 889.}	20.6293
31	R0	{2.46831, 8.14308}	{1.93258, 13.8204}	{{1.19018, 6.16453}, {2.85956, 19.5242}}	{26.8846, 78.2157}	inf	13.	164.751	{8.26673, 388.95}	1182.26	0.985779	newly	16.	116.41	{6., 283.}	649.313	0.842171	Reff	2.46792	1.8821	{1.15086, 2.7524}	27.969	ar	88 762.	64 221.5	{28 247., 92 658.}	28.9639
32	R0	{1.93236, 7.38987}	{1.99652, 17.2352}	{{1.49826, 9.70815}, {2.6579, 20.}}	{19.5588, 133.227}	inf	96.	333.805	{190.303, 588.248}	247.714	0.499341	newly	158.	174.59	{89., 273.}	42.5	0.169968	Reff	1.92828	1.97265	{1.48112, 2.6504}	19.7706	ar	77 654.	77 369.5	{61 349., 93 588.}	14.0388
33	R0	{2.82251, 17.6954}	{1.77348, 16.8891}	{{1.45025, 12.5218}, {2.0105, 20.}}	{37.1665, 14.8657}	inf	586.	451.746	{275.36, 603.54}	34.586	0.181885	newly	325.	226.31	{111., 324.}	37.6277	0.221189	Reff	2.7977	1.74779	{1.43004, 1.99131}	37.5275	ar	92 745.	71 379.6	{56 963., 82 078.}	23.056
34	R0	{1.87083, 12.3994}	{1.81667, 19.5988}	{{1.65226, 19.5617}, {1.90711, 20.}}	{3.84717, 58.0627}	inf	1336.	1425.12	{1236.45, 1663.58}	10.952	0.0443112	newly	975.	585.88	{501., 697.}	39.9097	0.225505	Reff	1.81905	1.77514	{1.62042, 1.85701}	3.47767	ar	76 282.	78 216.5	{71 952., 81 700.}	5.64283
35	R0	{2.95779, 16.3003}	{1.89682, 18.731}	{{1.64477, 15.8591}, {2.11846, 20.}}	{35.8702, 17.3058}	inf	233.	441.914	{277.102, 607.9}	89.7105	0.257784	newly	159.	199.	{108., 278.}	41.761	0.15399	Reff	2.94682	1.87731	{1.64017, 2.08868}	36.2935	ar	93 873.	78 681.8	{69 568., 85 562.}	16.1992
36	R0	{2.18718, 14.261}	{1.92082, 17.677}	{{1.59465, 14.5262}, {2.1305, 20.}}	{12.1784, 25.5939}	inf	1258.	1066.5	{758.192, 1325.95}	17.9932	0.0920333	newly	857.	507.68	{396., 666.}	40.7608	0.23556	Reff	2.13862	1.87564	{1.58328, 2.07642}	12.2968	ar	84 379.	79 478.2	{68 232., 86 134.}	7.06385
37	R0	{2.44912, 9.31118}	{1.99529, 16.6269}	{{1.35191, 10.9997}, {2.96542, 20.}}	{25.611, 80.6374}	inf	41.	234.282	{90.7647, 417.827}	471.42	0.691819	newly	54.	128.08	{49., 216.}	145.074	0.341655	Reff	2.44728	1.96868	{1.34951, 2.95203}	25.8902	ar	88 442.	73 221.9	{48 296., 94 761.}	19.9746
38	R0	{2.05949, 5.72514}	{1.9693, 16.1454}	{{1.31606, 8.45307}, {2.71682, 20.}}	{24.1753, 182.009}	inf	11.	245.604	{111.049, 439.68}	2132.76	1.28205	newly	26.	137.92	{55., 235.}	430.923	0.652168	Reff	2.05842	1.93258	{1.31259, 2.68207}	24.1295	ar	81 587.	72 394.3	{45 558., 92 397.}	19.9408
39	R0	{1.75505, 8.52404}	{1.80919, 19.738}	{{1.69661, 19.8351}, {1.88667, 20.}}	{4.76095, 131.556}	inf	489.	815.152	{651.832, 957.087}	66.6977	0.216939	newly	574.	333.65	{266., 387.}	42.1829	0.244123	Reff	1.73539	1.79373	{1.68258, 1.8696}	4.96341	ar	70 925.	77 905.3	{73 733., 81 096.}	10.2183
40	R0	{2.24724, 13.7861}	{1.89752, 16.8179}	{{1.59394, 13.6697}, {2.13428, 20.}}	{15.9792, 25.3337}	inf	1281.	1092.73	{753.898, 1397.81}	21.5688	0.109495	newly	906.	549.51	{388., 728.}	40.043	0.236024	Reff	2.19627	1.85762	{1.54095, 2.09966}	15.8115	ar	85 257.	78 258.	{67 200., 86 179.}	8.91921
41	R0	{1.77635, 8.62406}	{1.92498, 17.0981}	{{1.61843, 13.6848}, {2.16502, 20.}}	{14.611, 98.26}	inf	634.	843.769	{574.97, 1017.12}	37.6856	0.124515	newly	682.	419.43	{274., 563.}	42.0953	0.250289	Reff	1.75212	1.88257	{1.57698, 2.1307}	13.8338	ar	72 941.	78 897.3	{69 012., 86 774.}	11.643
42	R0	{1.80397, 5.4932}	{2.05929, 16.213}	{{1.36882, 11.2029}, {2.89241, 20.}}	{26.9699, 195.146}	inf	36.	257.031	{99.8789, 420.506}	613.975	0.782596	newly	83.	148.68	{53., 234.}	96.9639	0.267504	Reff	1.80242	2.03163	{1.35415, 2.88253}	26.6569	ar	73 232.	75 544.	{49 188., 94 254.}	20.0319
43	R0	{2.83096, 16.7077}	{1.78468, 20.}	{{1.72928, 20.}, {1.83722, 20.}}	{36.9586, 19.7053}	inf	491.	806.302	{669.226, 945.245}	64.2163	0.211238	newly	311.	322.41	{265., 377.}	12.582	0.052659	Reff	2.80905	1.76591	{1.71478, 1.81827}	37.1349	ar	92 740.	76 963.7	{74 864., 79 172.}	17.0113
44	R0	{2.73678, 11.6021}	{2.07061, 17.7059}	{{1.49338, 13.2197}, {2.61857, 20.}}	{24.9977, 53.9965}	inf	128.	339.664	{179.378, 560.097}	166.31	0.38402	newly	125.	169.57	{90., 254.}	52.568	0.178107	Reff	2.73147	2.05298	{1.46778, 2.60993}	25.4633	ar	92 003.	79 451.1	{59 515., 93 675.}	14.4269
45	R0	{2.14235, 6.98601}	{1.89638, 17.9987}	{{1.54258, 14.5928}, {2.26112, 20.}}	{16.2165, 157.639}	inf	49.	333.716	{183.577, 520.376}	581.052	0.80031	newly	74.	157.77	{78., 243.}	114.662	0.294138	Reff	2.14079	1.87806	{1.53593, 2.24229}	16.8194	ar	82 974.	76 489.8	{63 466., 87 990.}	10.3749
46	R0	{2.23409, 9.21289}	{1.88952, 18.0775}	{{1.52703, 13.6497}, {2.56203, 20.}}	{19.7528, 96.4226}	inf	58.	331.955	{168.926, 554.866}	472.336	0.719084	newly	78.	159.99	{65., 291.}	111.295	0.28662	Reff	2.23183	1.86265	{1.49606, 2.54533}	20.6593	ar	85 163.	75 695.3	{61 489., 91 860.}	13.435
47	R0	{2.12258, 8.15265}	{2.00155, 16.1754}	{{1.37357, 8.47281}, {2.92741, 20.}}	{23.2545, 99.0901}	inf	73.	235.728	{93.2483, 422.727}	224.059	0.449864	newly	100.	133.97	{51., 230.}	60.45	0.22185	Reff	2.11978	1.97765	{1.36748, 2.91487}	23.1304	ar	82 690.	73 980.4	{50 884., 94 603.}	18.002
48	R0	{2.71771, 10.4636}	{2.02387, 16.5264}	{{1.3704, 11.1638}, {2.96479, 20.}}	{28.0506, 60.9174}	inf	20.	236.831	{86.0597, 417.827}	1084.15	1.00637	newly	23.	133.89	{46., 224.}	482.217	0.679859	Reff	2.71654	1.99404	{1.36576, 2.93311}	28.8336	ar	91 716.	74 831.1	{49 813., 94 672.}	19.3928
49	R0	{2.44369, 6.66477}	{1.92059, 13.8861}	{{1.18976, 6.16453}, {2.75598, 19.5242}}	{26.6017, 112.428}	inf	7.	171.233	{16.5687, 388.95}	2357.59	1.22894	newly	14.	118.7	{6., 271.}	766.714	0.892889	Reff	2.44335	1.8705	{1.15086, 2.7524}	27.8118	ar	88 827.	63 558.8	{17 527., 91 955.}	29.6445
50	R0	{2.77383, 17.9775}	{1.80861, 19.9272}	{{1.71672, 19.8038}, {1.87187, 20.}}	{34.7973, 11.0055}	inf	1401.	985.121	{828.581, 1168.21}	29.704	0.157251	newly	799.	395.64	{323., 485.}	50.4831	0.310462	Reff	2.71353	1.78528	{1.69372, 1.84716}	34.2082	ar	92 334.	78 077.	{74 506., 80 554.}	15.4407
